# Crp and Arc system directly regulate the transcription of NADH dehydrogenase genes in *Shewanella oneidensis* nitrate and nitrite respiration

**DOI:** 10.1128/spectrum.03324-24

**Published:** 2025-05-16

**Authors:** Jia-Rong Liu, Zhi-Qing Wang, Fei-Fei Li, Zhen-Kun Li, Ming-Chen Wang, Na Wang, Yu An, Xiu-Lan Chen, Yu-Zhong Zhang, Hui-Hui Fu

**Affiliations:** 1 MOE Key Laboratory of Evolution and Marine Biodiversity, Frontiers Science Center for Deep Ocean Multispheres and Earth System & College of Marine Life Sciences, Ocean University of China 12591, Qingdao, China; 2 Laboratory for Marine Biology and Biotechnology, Qingdao Marine Science and Technology Center, Qingdao, China; 3 State Key Laboratory of Microbial Technology, Marine Biotechnology Research Center, Shandong University 12589, Qingdao, China; 4 Joint Research Center for Marine Microbial Science and Technology, Shandong University and Ocean University of China, Qingdao, China; Michigan State University, East Lansing, Michigan, USA

**Keywords:** NADH dehydrogenases, respiratory chain, global regulator, *Shewanella oneidensis*

## Abstract

NADH oxidation by NADH dehydrogenases (NDHs) is crucial for feeding respiratory quinone pool and maintaining the balance of NADH/NAD^+^. In the respiratory model organism *Shewanella oneidensis*, which possesses four NDHs, the longstanding notion had been that NDHs were not required under anoxic conditions until recent studies demonstrated their role in extracellular electron transfer. However, the role of each NDH, particularly under anoxic conditions, has not been characterized. Here, we systematically investigated the role of each NDH in aerobic and anaerobic nitrate and nitrite respiration using NDH triple mutants. We corroborated the involvement of NDHs in anaerobic nitrate/nitrite respiration, revealing different repertoires of NDHs employed by *S. oneidensis* in response to electron acceptor (EA) availability. The transcript levels of two *nqrs* were modulated by the EA conversion from nitrate to nitrite. Furthermore, we demonstrated that the global regulators Crp and the Arc system both directly controlled the transcription of four NDHs during nitrate/nitrite respiration. This study confirms the requirement of NDHs for anaerobic nitrate and nitrite respiration and sheds light on the respiratory remodeling mechanism whereby global regulators coordinate NDH genes transcription to adapt to redox-stratified environments.

**IMPORTANCE:**

NADH is an important electron source for the respiratory quinone pool. Multiple NADH dehydrogenases (NDHs) are widely present in prokaryotes, indicating the flexibility in NADH oxidation. As a renowned respiratory versatile model strain, *Shewanella oneidensis* possesses four NDHs, encompassing all three types of NDHs, with varying ion-translocating efficiencies. The redundancy of NDHs may confer advantages for *S. oneidensis* to survive and thrive in redox-stratified environments. However, the roles of each NDH, especially in anaerobic respiration, are less understood. Here, we evaluated the role of each NDH in aerobic and anaerobic nitrate/nitrite respiration. We found that the conversion of electron acceptor from nitrate to nitrite triggered the changes in the transcriptional levels of NDH genes, and global regulators Crp and the Arc system were involved in these processes. These findings elucidate the mechanism of the respiratory chain remodeling at the NADH oxidation step in response to different electron acceptors.

## INTRODUCTION

Members of genus *Shewanella*, belonging to γ-proteobacteria, are known for their ability to respire a diverse range of electron acceptors (EAs), including oxygen, nitrate, nitrite, fumarate, dimethylsulfoxide, trimethylamine *N*-oxide, iron, manganese oxides, and electrodes in microbial fuel cells (1–3). Extensive studies have characterized the respiratory chains from quinol pool to terminal EAs using the model strain *Shewanella oneidensis* MR-1 (2, 4–12). However, the upstream of the respiratory chains, where electrons are transferred into the quinone pool, remains poorly understood.

NADH is an important electron carrier in respiratory chains. It must be oxidized into NAD^+^ by NADH dehydrogenase (NDH) to feed electrons into the respiratory quinone pool and maintain the balance of NADH/NAD^+^, which is crucial for cell survival (13–16). Unlike mammals, which contain only one Nuo in their mitochondrial electron transport chain, prokaryotes often possess multiple NDHs (14, 15, 17–22). The genome of *S. oneidensis* MR-1 encodes four NDHs: one proton-pumping Nuo (4H^+^/2e^−^), two sodium-pumping Nqrs (2Na^+^/2e^−^), and one “uncoupling” Ndh (14–16, 18, 23, 24). Among these, Nuo is the most efficient, as the transfer of 2 e^−^ from NADH to quinone is coupled with the translocation of 4 H^+^ across the inner membrane, contributing to both ATP production and membrane potential maintenance (13, 25, 26). This redundancy of NDHs may facilitate the survival of *S. oneidensis* MR-1 by optimizing its respiratory chains in redox-stratified environments.

Respiratory chain remodeling at the NADH oxidation step has been reported in several prokaryotes (13, 14, 27–31). Duhl et al. (32) recently demonstrated that either Nuo or Nqr1 is essential for aerobic growth in *S. oneidensis* MR-1 by generating the single and double NDH mutants. Subsequent study revealed that the aerobic growth defect of the Δ*nuo*Δ*nqr1* mutant may derive from TCA cycle inhibition caused by an elevated NADH/NAD^+^ ratio (25). Nevertheless, the specific roles of individual NDHs in aerobic respiration are still unclear. Under anoxic conditions, *S. oneidensis* MR-1 primarily generates ATP through substrate-level phosphorylation via respiratory chains, with anaerobic lactate catabolism being independent of NADH-dependent enzymes, such as NDHs and pyruvate dehydrogenase (33–37). However, Hirose et al. (38) recently reported that the deletion of all four NDH complexes (ΔNDH mutant) almost completely abolished the ability of current generation of *S. oneidensis* MR-1 in the presence of a high-potential electrode (+0.5 V) and significantly reduced nitrate and MnO_2_ reduction capabilities, while fumarate respiration remained unaffected. Following this study, Madsen et al. (26) demonstrated Nuo and Nqr1—previously identified as essential for aerobic growth (32)—also contribute to extracellular electron transfer using either anode (+0.5 V) or Fe^3+^-NTA as EAs. These results collectively demonstrate the involvement of NDHs in anaerobic respiration of certain EAs.

Transcriptomic analyses of *S. oneidensis* have revealed the transcriptional changes of NDH genes depending on growth conditions (39, 40). These NDH genes are predicted to belong to the regulons of global regulators including Crp, HexR, and EtrA (41–44). Crp, essential for anaerobic respiration in *S. oneidensis* MR-1, is required for growth on diverse EAs (45, 46). The *crp* mutation significantly reduces transcription of key anaerobic respiration genes, including the nitrate reductase gene operon *nap*, nitrite reductase gene *nrfA,* and the two-component system *narPQ* (47). Crp-binding sites are predicted in promoter regions of these genes as well as in the DMSO reductase operon (*dms*) and *cymA*, which encodes a quinol oxidase shared by multiple anaerobic respiratory pathways (48). Notably, potential Crp-binding sites have been identified in the promoter regions of *nuoA* and *nqrA1* (42), suggesting that the globally regulatory effect of Crp may also derive from its regulation on NADH oxidation. The Arc system, a global respiratory regulator that senses the redox state of inner membrane quinone (49), plays critical roles in both aerobic and anaerobic DMSO respiration in *S. oneidensis* (42, 47, 50, 51). Despite sharing 73.6% amino acid sequence identity with *E. coli* Fnr—a master regulator of the aerobic-anaerobic metabolism switch—extensive experimental evidence demonstrates that EtrA exerts no significant regulatory influence on respiratory pathways in *S. oneidensis* MR-1 (42, 52). An ArcA-binding site is predicted in the *dms* operon, and the *arcA* mutant showed reduced *dms* expression (51). Recent study demonstrates that ArcA directly regulates the transcription of *nuo* during electrode respiration (38), promoting us to investigate whether the Arc system regulates NDHs during respiration on the other EAs.

**Fig 1:**
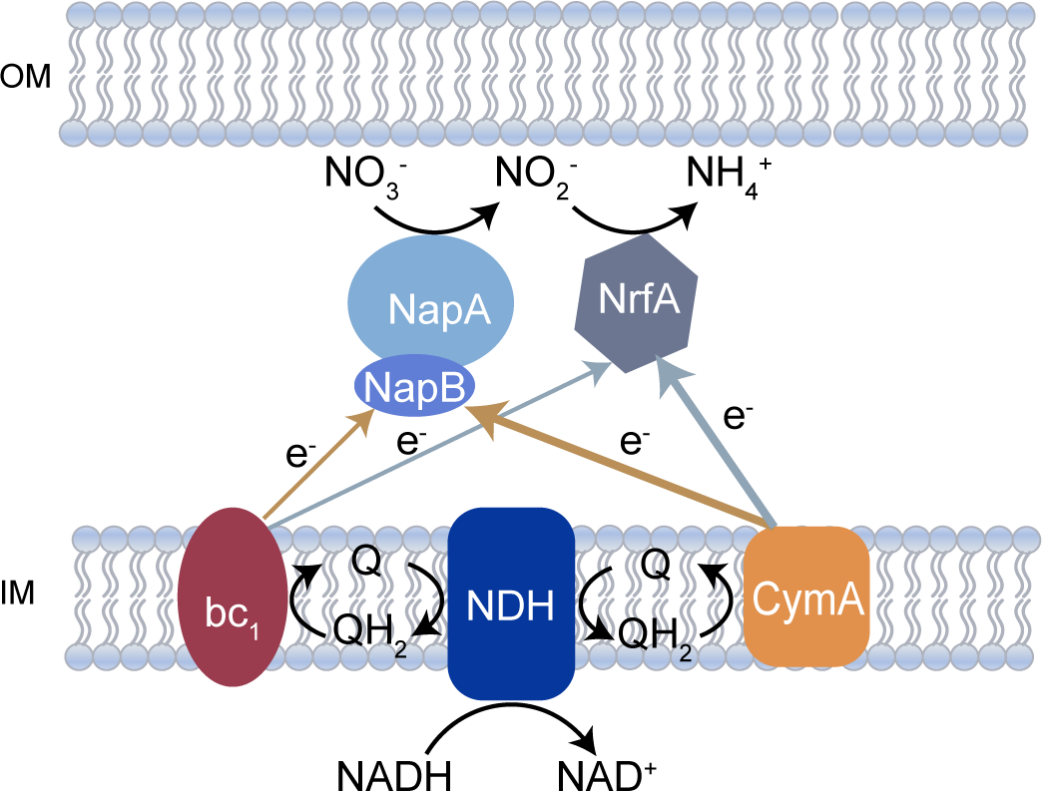
Nitrate and nitrite respiratory chains in *S. oneidensis* MR-1. Electron transfer pathways are color-coded based on respiratory conditions: brown arrows indicate the preferred electron flow during nitrate respiration; light slate gray arrows represent the electron flow during nitrite respiration. Thick arrows denote the primary electron transport mediated by CymA from the quinol pool to nitrate/nitrite reductases, while thin arrows illustrate secondary electron transfer via the cytochrome *bc*_1_ complex. OM, outer membrane; IM, inner membrane.

Nitrate and nitrite serve as common EAs for *S. oneidensis* MR-1 via respiratory nitrate ammonification (Fig. 1) (5, 6, 53). The impaired growth of ΔNDH mutant under nitrate-reducing conditions suggests the involvement of NDHs in nitrate and nitrite respiration (38). Notably, the electron transport components from the quinol pool to terminal reductases are identical for both nitrate and nitrite reduction (5, 6). Therefore, the differences in NDH mutants must stem from the absence of certain NDH(s) rather than electron transfer efficiency or preference. The most intriguing feature of respiratory nitrate ammonification in *S. oneidensis* is that nitrite reduction does not occur until nitrate is thoroughly consumed (6). This inherent EA conversion during complete nitrate respiration makes *S. oneidensis* an ideal model for investigating respiratory chain remodeling mechanisms in response to different EAs.

This study aimed to elucidate the respiratory remodeling mechanism at the NADH oxidation step during anaerobic nitrate and nitrite respiration in the model strain *S. oneidensis*. To this end, NDH triple mutants (each remaining only one NDH) were generated and characterized under aerobic and anaerobic nitrate and nitrite respiration conditions. Transcriptional analysis showed that NDH genes responded to the change of available EAs, especially the two *nqrs* exhibited opposing transcriptional responses during the EA conversion from nitrate to nitrite. Furthermore, we demonstrated that both Crp and the Arc system directly regulate the transcription of all four NDHs during nitrate and nitrite respiration. This study characterized the roles of individual NDHs under aerobic and anaerobic conditions and elucidated the mechanism by which the global regulators Crp and Arc coordinate NDH transcription in response to the availability of EAs in the environments.

## MATERIALS AND METHODS

### Strains, media and culture conditions

Bacterial strains and plasmids used in this study are listed in Table S1. Routinely, *Escherichia coli* and *S. oneidensis* strains were grown in Lysogeny broth (LB) at 37°C and 30°C, respectively, for genetic manipulation. When appropriate, the growth medium was supplemented with the following: 2,6-diaminopimelic acid (DAP), 0.3 mM; gentamicin, 15 µg/mL; kanamycin, 50 µg/mL; ampicillin, 50 µg/mL. All growth experiments utilized the defined MS minimal medium with lactate added to a final concentration of 20 mM as electron donor, which has been widely used for physiological characterization of *S. oneidensis* MR-1 under both oxic and anoxic conditions (54–56). For anaerobic growth, exponential-phase cultures initially grown aerobically were centrifuged and inoculated into fresh defined MS medium [KCl, 1.34 mM; NaH_2_PO_4_, 5 mM; Na_2_SO_4_, 0.7 mM; NaCl, 52 mM; piperazine-*N*,*N*′-bis(2-ethanesulfonic acid) (PIPES), 3 mM; NH_4_Cl, 28 mM; sodium lactate, 20 mM; MgSO_4_, 1 mM; CaCl_2_, 0.27 mM; and FeCl_2_, 3.6 µM, pH 7.0] in the Hungate tubes to an OD_600_ of ~0.01. The cultures were purged with nitrogen for ~1 h in the anaerobic chamber until the oxygen content dropped below 5 ppm (57). EAs used in this study included nitrate (5 mM), nitrite (5 mM), and fumarate (20 mM).

### In-frame deletion and complementation

In-frame deletion strains were constructed using the *att*-based fusion PCR method, as described previously (58). In brief, the flanking fragments of the target gene were amplified by PCR with *attB-*containing primers and linked by a second round of PCR (Table S2). The fused fragment was cloned into plasmid pHGM01 (58) using the Gateway BP Clonase II enzyme mix (Invitrogen) following the manufacturer’s instruction and maintained in *E. coli* WM3064. The resultant plasmid was transferred into *S. oneidensis* via conjugation: Donor cells (*E. coli* WM3064) and recipient cells (*S. oneidensis* MR-1) were separately grown in LB medium to an OD_600_ ≈ 0.4–0.6, mixed at a 2:1 (vol/vol) ratio and spotted onto LB agar plates supplemented with 0.3 mM DAP. The correct conjugants were selected by gentamycin resistance and verified by PCR. Subsequently, verified conjugants were cultured in LB broth lacking NaCl and plated onto LB agar containing 10% (wt/vol) sucrose. Target gene-deletion mutants were screened for gentamicin sensitivity and sucrose resistance, followed by confirmation through DNA sequencing. For the generation of Δ*nuoN*Δ*ndh*Δ*nqrF1*, conjugation and the following screening procedures were performed anaerobically with fumarate and lactate supplemented.

For genetic complementation, the target genes were amplified from the genomic DNA of *S. oneidensis* MR-1 using specific primers (Table S2) and subsequently cloned into the plasmid pHGE-P*_tac_* under the control of isopropyl β-D-1-thiogalactopyranoside (IPTG)-inducible P*_tac_* promoter (59). The complementation vector was transferred into the corresponding mutant strain via conjugation and verified by PCR and sequencing.

### Analysis of the nitrate and nitrite utilization abilities

Strains cultured in defined MS medium with 5 mM nitrate or nitrite as the sole EA under anoxic conditions were sampled at indicated time points. Nitrate and nitrite concentrations in the culture supernatants were quantified using ion chromatography (IC) as previously described (6). The concentrations of nitrite in culture were also measured by a modified Griess assay (60). In all assays, a nitrite standard curve was generated for each assay, and culture samples were diluted appropriately if necessary.

### Cell density measurement

The wild-type and four triple mutant strains were anaerobically cultured in defined MS medium with nitrite (5 mM) as the sole EA for 78 h. Cells were harvested and fixed using 5% (vol/vol) glutaraldehyde and then kept in the dark for 20 min. Cell densities were measured using a Guava EasyCyte HT flow cytometer equipped with Guava InCyte v3.1 software, following the instrument configuration described by He et al. (61). After staining with SYBR Green I (Solarbio) for 40 min at room temperature in the dark, cells were serially diluted. An appropriate dilution (1:100, yielding 10^5^–10^6^ cells/mL) was selected for final cell density calculations.

### Real-time qPCR analysis

For aerobic RT-qPCR, bacterial cultures were pre-grown overnight in LB medium, then inoculated into fresh LB medium and incubated at 30°C to OD_600_ ~ 0.6. Cells were subsequently harvested for total RNA extraction. To anaerobic RT-qPCR, bacteria were pre-cultured in LB medium to the mid-exponential phase, centrifuged, and transferred into nitrogen-purged defined MS medium with 20 mM lactate as the electron donor and 5 mM nitrate or nitrite as the sole electron acceptor, respectively. Cells were incubated under anoxic conditions for 2 hours before RNA extraction unless otherwise noted. To assess the transcriptional response of NDH genes to EA conversion, wild-type *S. oneidensis* MR-1 was cultured anaerobically in defined MS medium with 5 mM nitrate as the sole EA and harvested at indicated time points. Total RNA was extracted using the RNeasy Mini Kit (Qiagen, Germany) following the manufacturer’s instructions and transcribed into cDNA using a PrimeScript RT reagent Kit with gDNA Eraser (TaKaRa, Japan). The qPCR was performed with specific primers (Table S2) using SYBR Premix Ex Taq (TaKaRa, Japan). Relative gene expression levels were normalized to the housekeeping gene *recA* using the 2^-ΔΔCt^ method.

### Gene cloning and protein expression and purification

The *crp* and *arcA* genes were amplified from the *S. oneidensis* MR-1 genome using primers listed in Table S2. The *crp* gene was cloned into the pMAL-c4x vector (NEB, England) to generate an N-terminal maltose-binding protein (MBP)-tagged Crp, while *arcA* was cloned into the pET22b vector (Novagen, Germany) to generate a C-terminal 6 × His-tagged ArcA. For microscale thermophoresis (MST) assays, an additional 6 × His tag was introduced at the C-terminus of Crp via PCR to facilitate the fluorescent labeling (see MST-binding assay section). MBP-Crp and His-tagged ArcA were heterologously expressed in *E. coli* BL21(DE3) by induction with 0.5 mM IPTG. Cells were harvested via centrifugation and resuspended in lysis buffer [50 mM Tris-HCl, 100 mM NaCl, 0.5% (vol/vol) glycerol, pH 8.0]. Cells were disrupted by pressure crusher. MBP-Crp was purified by affinity chromatography using a maltose-column (Cytiva, Sweden), whereas ArcA was purified using a Ni^2+^-NTA column (GE Healthcare, America). The eluents of MBP-Crp and ArcA were further fractionated by gel filtration on a Superdex G200 column (GE Healthcare, America). As a negative control, the MBP tag alone was amplified from pMAL-c4x vector and purified as MBP-Crp.

### Electrophoretic mobility shift assays

EMSAs were performed with purified MBP-Crp or ArcA and biotinylated DNA probes (10 nM) in binding buffer [2 mM EDTA, 20 mM KCl, 0.5 mM dithiothreitol (DTT), 4% (wt/vol) Ficoll-400, pH 8.0] with 2 µg poly (dI-dC) as a nonspecific competitor (62). The promoter regions of NDH operons were amplified using a specific primer set, with the forward primer 5′-end labeled with biotin (Table S2). For reactions involving MBP-Crp, 10 µM cyclic adenosine monophosphate (cAMP) was added to the mixture. To rule out nonspecific interactions between the MBP tag and DNA probes, a control reaction containing purified MBP (instead of MBP-Crp) was included and electrophoresed on the same non-denaturing polyacrylamide gel. Prior to DNA-binding assays, ArcA was phosphorylated *in vitro* using established protocols (5, 50). Competitive EMSAs were conducted by adding a 50-fold molar excess of unlabeled DNA probe to the reaction mixture.

### Microscale thermophoresis-binding assay

MBP-Crp (cAMP-activated) or phosphorylated ArcA were labeled with the Large Volume Protein Labeling Kit RED-Tris-NTA 2nd Generation (NanoTemper Technologies GmbH). DNA ligands were serially diluted in a range of concentration and incubated with labeled proteins at 25°C in binding buffer containing 1 × PBS (pH 7.4) and 0.05% (vol/vol) Tween-20. Reaction mixtures were loaded into MonolithTM NT.115 Series capillaries (NanoTemper Technologies GmbH) and analyzed by MST with the following parameters: 60% LED power and “medium” MST power. The MST data were fit in the MO.Affinity Analysis software to yield the *K*_d_ values.

## RESULTS

### Characterization of NDH mutants in aerobic respiration

**Fig 2:**
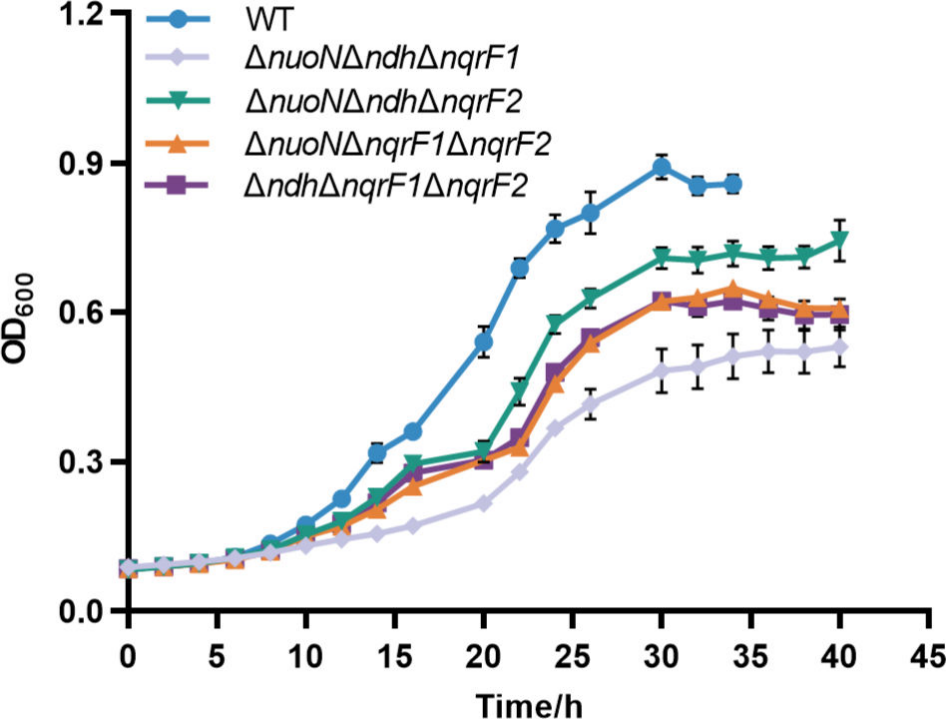
Aerobic growths of wild type and NDH triple mutants. Growth of wild-type *S. oneidensis* MR-1, Δ*nuoN*Δ*ndh*Δ*nqrF1*, Δ*nuoN*Δ*ndh*Δ*nqrF2*, Δ*nuoN*Δ*nqrF1*Δ*nqrF2,* and Δ*ndh*Δ*nqrF1*Δ*nqrF2* strains in defined MS medium with lactate (20 mM) as sole electron donor in aerobic condition. The error bar represents the standard deviation of triplicate experiments. All experiments were carried out at least three times.

To characterize the function of each NDH, triple NDH knockout strains were generated, which only retained one functional NDH. We made in-frame deletion of an essential gene of each NDH operon, *nuoN* (SO_1009), *ndh* (SO_3517), *nqrF1* (SO_1108), and *nqrF2* (SO_0907), as Duhl et al. (32) did. This generated four single mutants, six double mutants, and four triple mutants. Notably, the Δ*nuoN*Δ*ndh*Δ*nqrF1* mutant could only be obtained under anoxic conditions with fumarate as the EA, reminiscent of the aerobic growth defect observed in ΔNDH mutant (38). This implies a negligible role of Nqr2 in aerobic respiration. Among these single and double mutants, only Δ*nuoN*Δ*nqrF1* exhibited severe growth defects in minimal medium with lactate as the sole carbon and energy source under oxic conditions (Fig. S1A and B), consistent with Duhl et al. (32). When any three NDHs were knocked out simultaneously, pronounced growth defects appeared under oxic conditions (Fig. 2). Among them, the growth rate of Δ*nuoN*Δ*ndh*Δ*nqrF1* was lowest (*μ* = 0.092 ± 0.005 h^−1^), implying the least contribution of Nqr2 in aerobic NADH oxidation. Meanwhile, Δ*nuoN*Δ*ndh*Δ*nqrF2* had the most robust growth (*μ* = 0.121 ± 0.009 h^−1^) compared with the other triple mutants (Δ*nuoN*Δ*ndh*Δ*nqrF1*: *μ* = 0.092 ± 0.005 h^−1^; Δ*nuoN*Δ*nqrF1*Δ*nqrF2*: *μ* = 0.119 ± 0.007 h^−1^; Δ*ndh*Δ*nqrF1*Δ*nqrF2*: *μ* = 0.115 ± 0.005 h^−1^), implying that Nqr1 was the predominant NDH in aerobic respiration. This is consistent with the much higher transcript level of *nqrA1* compared with the other NDH genes under oxic conditions (Fig. S1C). Sodium-pumping NDHs are conserved across *Shewanella* spp., whereas proton-pumping NDHs are sporadically distributed (Table S3). This suggests that Nqrs serve as primary aerobic NDHs in most *Shewanella* members despite that Nuo is more efficient. It is interesting to study the advantage or necessity to remain Nuo in some *Shewanella* strains.

### Nitrate and nitrite respiration of NDH triple mutants under anoxic conditions

**Fig 3:**
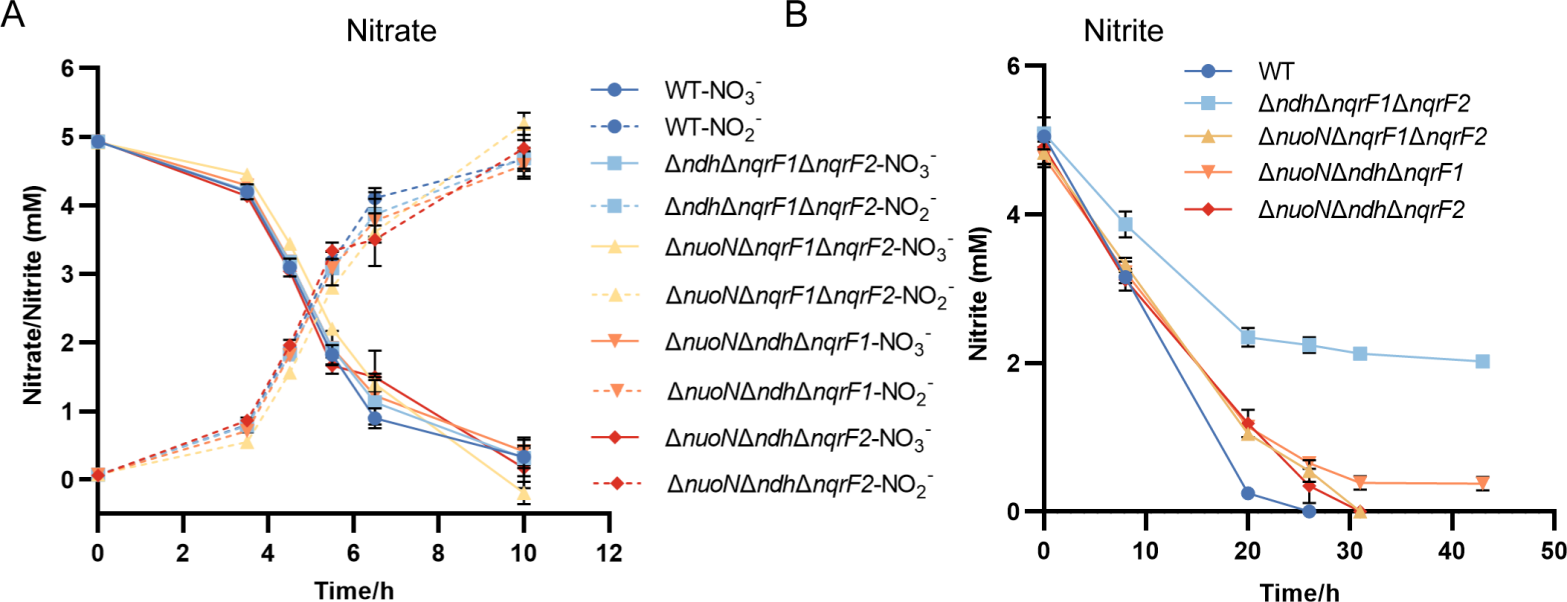
Consumption of nitrate and nitrite by wild type and NDH triple mutants under anoxic condition. Nitrate and nitrite utilization of wild-type *S. oneidensis* MR-1, Δ*nuoN*Δ*ndh*Δ*nqrF1*, Δ*nuoN*Δ*ndh*Δ*nqrF2*, Δ*nuoN*Δ*nqrF1*Δ*nqrF2,* and Δ*ndh*Δ*nqrF1*Δ*nqrF2* strains in defined MS medium with lactate (20 mM) as sole electron donor and 5 mM nitrate (A) or nitrite (B) as sole EA, respectively, under anoxic conditions. Cultures were sampled at indicated times for nitrate and nitrite detection. The concentrations of nitrate and nitrite reduced from nitrate were shown simultaneously when nitrate was the EA. The error bar represents standard deviation of triplicate experiments. All experiments were carried out at least three times.

*S. oneidensis* employs a two-step nitrate respiration pathway: nitrate is initially reduced to nitrite, which can also serve as an EA of *S. oneidensis*, and only when nitrate is thoroughly consumed does the reduction of nitrite to ammonium occur (Fig. 1) (5, 6). Previous studies indicate that ΔNDH exhibited impaired nitrate utilization (38), implying the involvement of NDHs in anaerobic nitrate respiration. However, the observed growth defect of ΔNDH could derive from functional deficiencies in either or both steps of the pathway. To determine the exact roles of NDHs in each step, we quantified the concentrations of nitrate during anaerobic cultivation of wild-type MR-1 and four NDH triple mutant strains in minimal medium containing nitrate (5 mM) as the sole EA (50). All four triple mutants displayed nitrate consumption rates comparable to wild type (Fig. 3A), implying that the absence of any three NDHs barely impairs the nitrate respiration to nitrite. There are two possible explanations for this: one is that any NDH alone is sufficient to meet NADH oxidation requirements for nitrate to nitrite reduction; the other is that nitrate reduction to nitrite proceeds independently of NDHs. Therefore, ΔNDH is needed to distinguish between these possibilities.

When nitrate was replaced by nitrite, all triple mutants exhibited impaired nitrite utilization, with Δ*ndh*Δ*nqrF1*Δ*nqrF2* showing the most severe defect (Fig. 3B). Consistently, the maximum biomass yield supported by nitrite respiration was lowest in Δ*ndh*Δ*nqrF1*Δ*nqrF2* compared to the wild-type and other triple mutants (Fig. S2). These results demonstrate that the most efficient NDH, Nuo, contributes minimally to nitrite respiration. This physiologically distinction aligns with the markedly lower biomass yield from anaerobic nitrite respiration compared to aerobic respiration. Since nitrite is generally cytotoxic, we cannot exclude the possibility that Nuo is more sensitive to nitrite toxicity than other NDHs. Collectively, our findings indicate that NDHs are involved in anaerobic nitrate respiration at least during the nitrite reduction step, and the relative contributions of individual NDHs varied significantly depending on the EAs utilized by *S. oneidensis*.

### Transcript levels of NDH genes respond to the conversion of EA from nitrate to nitrite

**Fig 4:**
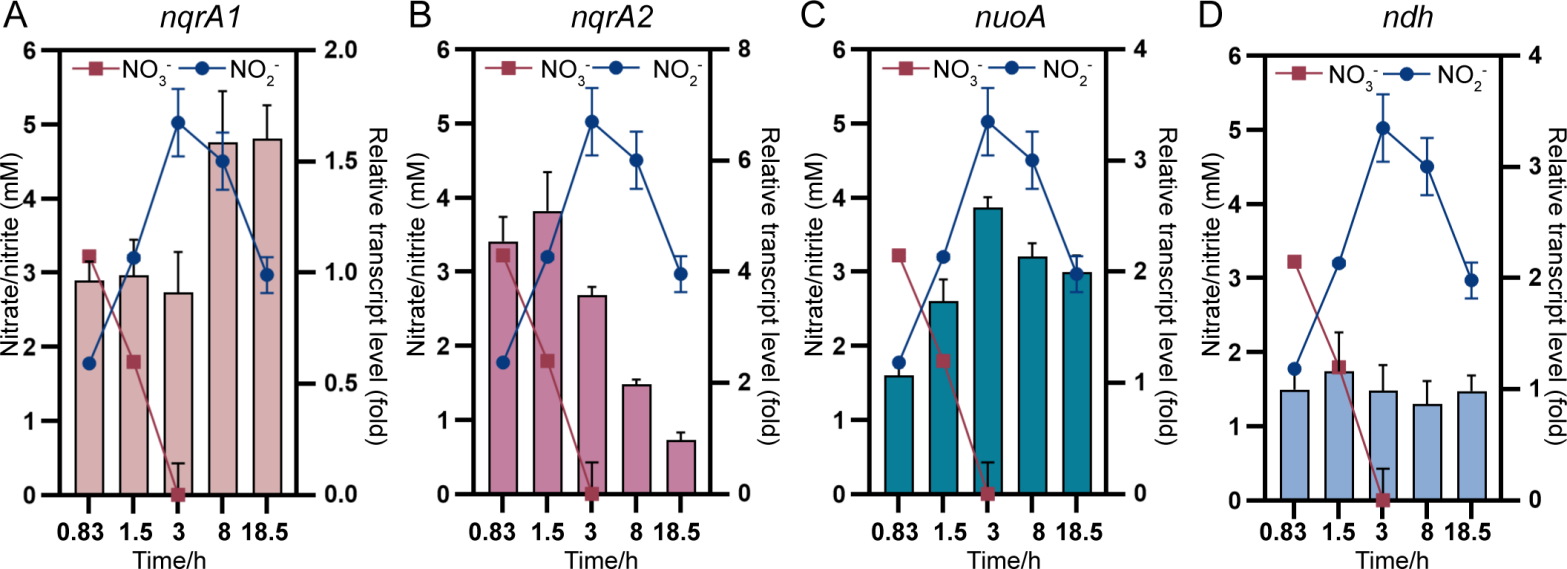
Changes in relative transcript levels of NDH genes in response to EA conversion from nitrate to nitrite under anoxic conditions. Relative transcript levels of *nqrA1* (A), *nqrA2* (B), *nuoA* (C), and *ndh* (D) in wild-type strain grown in defined MS medium with nitrate (5 mM) as sole EA in anaerobic condition. The nitrate and nitrite concentration curves were embedded to indicate the EA used at indicated sampling time points. The error bar represents the standard deviation of triplicate experiments. All experiments were carried out at least three times.

To determine whether *S. oneidensis* remodels its respiratory chain by regulating NDHs expression at the transcriptional level in response to different EAs, we measured the mRNA abundance of the first gene of each NDH operon in wild-type MR-1 during complete nitrate respiration. Mid-log phase cells (OD_600_ ~ 0.6) were inoculated into defined M5 medium with nitrate (5 mM) as the sole EA and harvested at designated time points. Nitrite (the nitrate reduction product) accumulated to ~5 mM, indicating complete nitrate reduction, before being subsequently consumed (Fig. 4).

Transcriptional analysis revealed that *nqrA1* transcription increased moderately (~1.6-fold) during nitrite respiration compared to nitrate respiration (Fig. 4A), while *nqrA2* transcription progressively decreased (up to ~5-fold) during the conversion of EA from nitrate to nitrite (Fig. 4B). These results imply that *S. oneidensis* differentially regulates the two *nqrs* depending on EA availability, potentially as an energy-conserving strategy. The transcript level of *nuoA* was gradually increased (~2.5-fold) with the accumulation of nitrite (Fig. 4C). However, the transcript levels of *nuoA* did not respond to nitrite concentration (Fig. S3). The *nuoA* transcription could not explain the limited contribution of Nuo to nitrite respiration yet (Fig. 3B). In contrast to these dynamic changes, *ndh* transcription remained relatively stable throughout the respiration process (Fig. 4D), implying the constitutive expression of the only one “non-ion-pumping” NDH in *S. oneidensis*. Collectively, these results demonstrate that the conversion of available EA significantly alters the transcript levels of most NDHs (except for *ndh*), which may contribute to respiratory remodeling at the NADH oxidation step.

### Crp and Arc system coordinate in modulating the transcription of two *nqrs*

The divergent transcriptional responses of the two homologous *nqr* operons to the conversion of EA from nitrate to nitrite promoted investigation into potential regulatory mechanisms. We focused on the global respiration regulators Crp and the Arc two-component system in *S. oneidensis*. Mutant strains Δ*crp* and Δ*arcA* (encoding the response regulator of the Arc system) (63) were generated to assess their roles in nitrate and nitrite utilization. Consistent with previous studies (42, 47), Δ*crp* barely utilized nitrate and nitrite (Fig. S4A and B). While Δ*arcA* exhibited only minor defects in nitrate and nitrite utilization (Fig. S4C and D), suggesting a limited role in nitrate and nitrite respiration.

**Fig 5:**
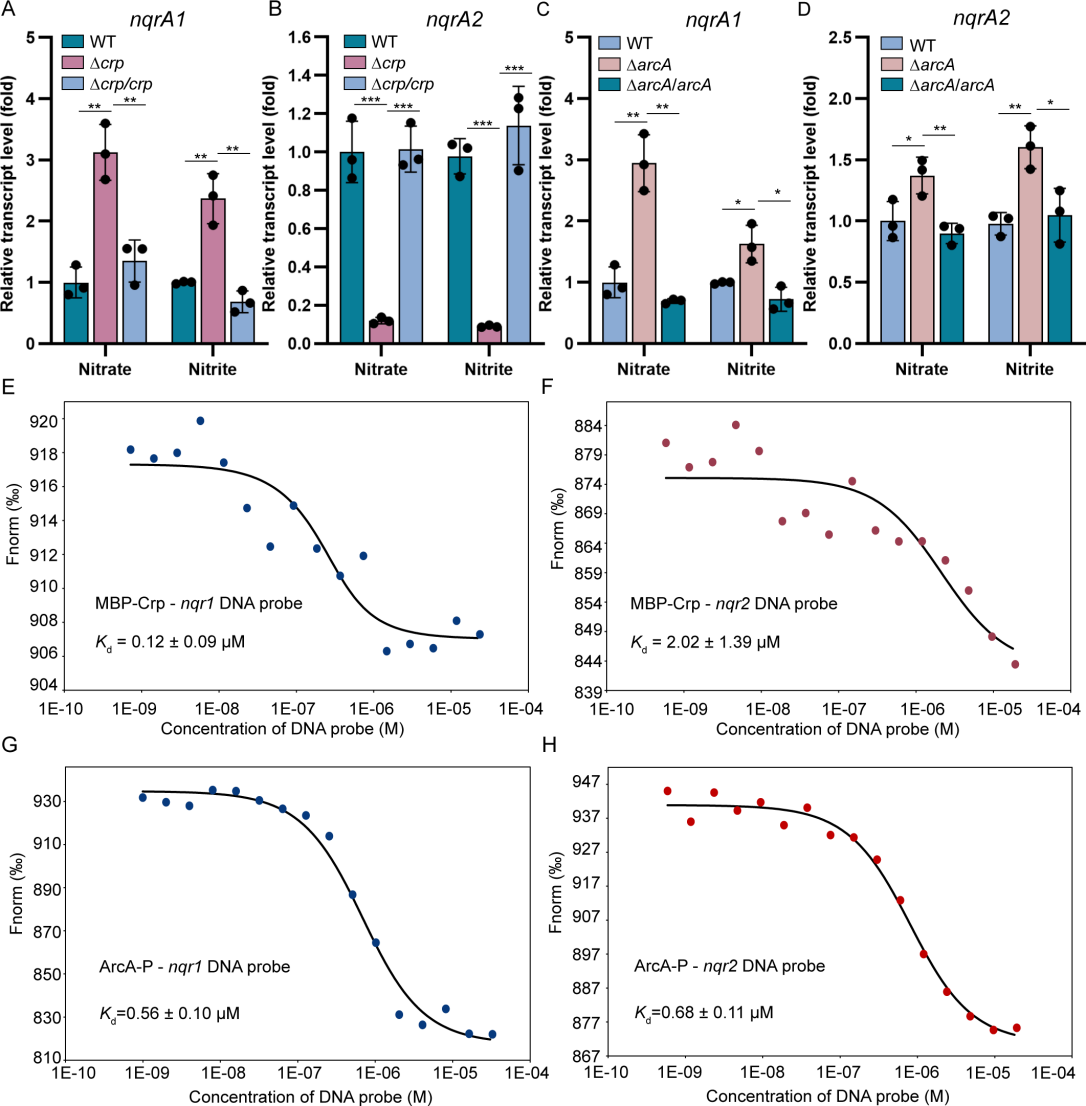
Crp and ArcA directly regulate the transcription of *nqr1* and *nqr2*. (A, B) Relative transcript levels of *nqrA1* (A) and *nqrA2* (B) in wild-type, Δ*crp,* and its genetically complemented strain Δ*crp*/*crp* grown in defined MS medium with 5 mM nitrate or nitrite as EA under anoxic conditions. The error bar represents standard deviation of triplicate experiments. (C, D). Relative transcript levels of *nqrA1* (C) and *nqrA2* (D) in wild-type, Δ*arcA,* and its genetically complemented strain Δ*arcA*/*arcA* grown in defined MS medium with 5 mM nitrate or nitrite as EA under anoxic conditions. The error bar represents standard deviation of triplicate experiments. (E, F) MST analysis of MBP-Crp binding to *nqr1* promoter probe (E) or *nqr2* promoter probe (F). (G, H) MST analysis of ArcA-P binding to *nqr1* promoter probe (G) or *nqr2* promoter probe (H). A two-sided Student’s *t*-test was used to assess statistically significant differences (^***^P < 0.001; ^**^P < 0.01; ^*^P < 0.05). All experiments were carried out at least three times.

To explore the involvement of Crp and ArcA in the divergent responses of two *nqrs*, the transcript levels of two *nqrAs* were measured in wild-type and mutant strains. Mid-log phase cultures (OD_600_ ~0.6) were transferred into anaerobic medium with either nitrate or nitrite supplemented as sole EA and harvested after 2 h (ensuring nitrate was the authentically utilized EA in nitrate-supplemented medium). The *nqrA1* transcript levels were increased 2.4- to 3.1-fold in Δ*crp* compared to wild type, and this enhancement was reduced in the presence of cloned *crp* (Fig. 5A). Conversely, *nqrA2* transcription decreased up to 8.3- to 11.1-fold in Δ*crp* compared to wild type, which was fully restored by cloned *crp* (Fig. 5B). This demonstrates Crp’s dual regulatory role as a repressor of *nqr1* but activator of *nqr2*. The deletion of *arcA* produced more modest effects, causing only ~1.5-fold expression changes except for *nqrA1* in nitrate-reducing condition (~3 fold) (Fig. 5C and D). This indicates ArcA acts as a repressor for two *nqrs*; however, its regulatory function is far less pronounced than that of Crp in modulating *nqr2* transcription.

To explore the regulatory mechanisms further, we purified recombinant MBP-tagged Crp and His-tagged ArcA. MST analysis demonstrated that cAMP-activated MBP-Crp had *K*_d_ values of 0.12 ± 0.09 µM and 2.02 ± 1.39 µM for *nqr1* promoter probe and *nqr2* promoter probe, respectively (Fig. 5E and F). Control experiments confirmed the specific nature of these interaction, as MBP alone could not bind to either probe (Fig. S5A and B). Consistently, the EMSA shift bands were detected for both probes incubated with cAMP-activated MBP-Crp but not with MBP control (Fig. S5C and D). These data, collectively, indicate that Crp directly regulates the transcription of two *nqrs*. Parallel analyses with phosphorylated ArcA (ArcA-P) revealed similar DNA-binding capability. MST yielded *K*_d_ values of 0.56 ± 0.10 µM and 0.68 ± 0.11 µM for ArcA-P binding to *nqr1* and *nqr2* promoters, respectively (Fig. 5G and H). Corresponding EMSA experiments showed specific protein-DNA complexes that were effectively competed by 50-fold excess unlabeled probes (Fig. S5E and F). These results indicate that both Crp and ArcA regulate the transcription of two *nqrs* by directly binding to their promoter regions.

### Crp and ArcA also directly regulate the transcription of *nuo* and *ndh*

**Fig 6:**
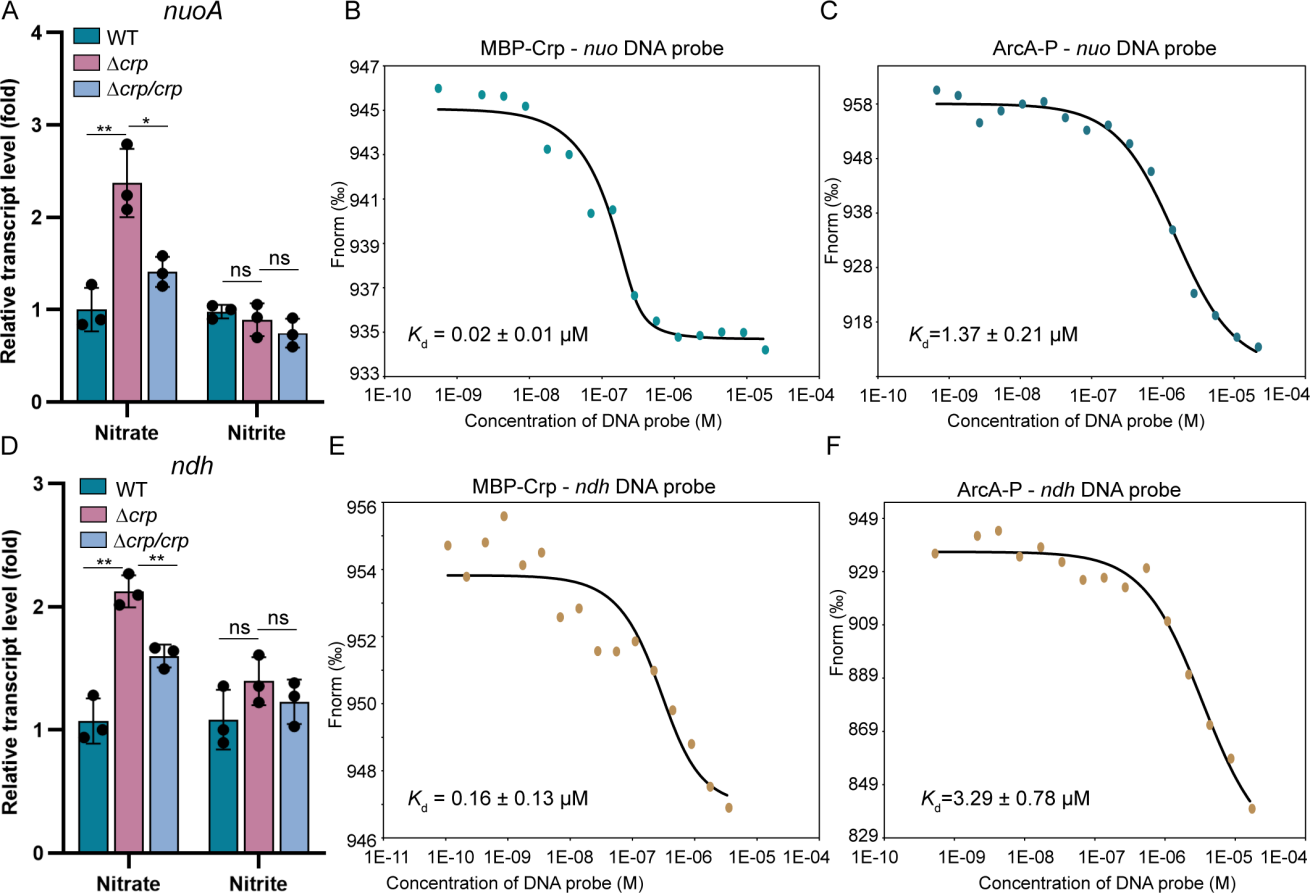
Crp and ArcA directly regulate the transcription of *nuo* and *ndh*. (A) Relative transcript levels of *nuoA* in wild-type, Δ*crp,* and its genetically complemented strain Δ*crp*/*crp* grown in defined MS medium with 5 mM nitrate or nitrite as EA under anoxic conditions. The error bar represents standard deviation of triplicate experiments. (B) MST analysis of MBP-Crp binding to *nuo* promoter probe. (C) MST analysis of ArcA-P binding to *nuo* promoter probe. (D) Relative transcript levels of *ndh* in wild-type, Δ*crp,* and its genetically complemented strain Δ*crp*/*crp* grown in defined MS medium with 5 mM nitrate or nitrite as EA under anoxic conditions. The error bar represents standard deviation of triplicate experiments. (E) MST analysis of MBP-Crp binding to *ndh* promoter probe. (F) MST analysis of ArcA-P binding to *ndh* promoter probe. A two-sided Student’s *t*-test was used to assess statistically significant differences (^**^P < 0.01; ^*^P < 0.05; ns, *P* > 0.05). All experiments were carried out at least three times.

Although transcript levels of *nuo* and *ndh* did not respond to changes in EA under complete nitrate respiration (Fig. 4C and D), we further examined the regulatory roles of Crp and ArcA on these operons. Under nitrate-reducing condition, the transcript level of *nuoA* in Δ*crp* increased moderately (~2.4-fold) compared to wild-type cells, an enhancement abolished by complementation with *crp* (Fig. 6A). However, under nitrite-reducing condition, the regulatory effect of *crp* on the *nuoA* was not significant (Fig. 6A). Potential Crp-binding site had been predicted in the promoter region of *nuoA* (42), implying direct regulation by Crp. To validate this, we performed MST and EMSA using the purified Crp and the *nuo* promoter DNA probe, following methods described for *nqrs*. As expected, interaction was observed between the *nuo* promoter probe and MBP-Crp, but not with MBP alone (Fig. 6B; Fig. S6A and B). These results indicate that Crp directly regulates the transcription of *nuo*.

Although ArcA has been reported to directly regulate *nuo* transcription during electrode respiration in *S. oneidensis* (38) and *E. coli* (64; Park et al. 2013), no significant regulatory effect of ArcA on *nuo* was observed under either nitrate- or nitrite-reducing condition (Fig. S6C). Nevertheless, consistent with previous findings (38), MST and EMSA confirmed interaction between ArcA-P and the *nuo* promoter probe (Fig. 6C; Fig. S6D). Thus, we speculate that the regulatory role of Δ*arcA* may be suppressed by Crp, which dominates transcriptional control during nitrate and nitrite respiration.

The absence of *crp* caused derepression of *ndh* under nitrate-reducing conditions (Fig. 6D). Like the other NDH genes, direct binding of MBP-Crp to the *ndh* promoter region was verified by both MST and EMSA (Fig. 6E; Fig. S6E and F), implying the involvement of *ndh* in the Crp regulon. In contrast, the deletion of *arcA* had negligible effects on the transcript levels of *ndh* in both nitrate- and nitrite-reducing conditions (Fig. S6G). Intriguingly, interactions between ArcA-P and the *ndh* promoter probe were still detected (Fig. 6F; Fig. S6H). Taken together, these results demonstrate that *nuo* and *ndh* are primarily regulated by Crp in nitrate and nitrite respiration, and the Arc system is also potentially involved.

## DISCUSSION

The long-standing assumption that NDHs are dispensable under anoxic conditions in *S. oneidensis* has been challenged by recent evidence demonstrating their role in high-potential electrode respiration (38). The *S. oneidensis* MR-1 genome encodes four NDHs spanning three distinct families, highlighting its flexibility in NADH oxidation. To date, only two studies have reported phenotypes associated with the double mutant Δ*nuoN*Δ*nqrF1*: impaired aerobic growth in minimal medium (32) and reduced current generation with high-potential electrodes (26). Notably, an apparent discrepancy exists between the Δ*nuoN*Δ*nqrF1* phenotype reported by Duhl et al. (32) and those observed in our study. While Duhl et al. described a complete loss of aerobic growth in M5 minimal medium, we observed only a significant growth defect under analogous conditions using defined MS medium (Fig. S1B). This divergence may stem from methodological differences, particularly in culture volume (300 µL vs 1 mL/50 mL) and medium composition (MS vs M5).

Critically, the individual contributions of NDHs have not been characterized yet, particularly under anoxic conditions. To address this gap, we generated four NDH triple mutants and systematically evaluated their roles in aerobic and anaerobic nitrate and nitrite respiration. Our findings reveal dynamic respiratory chain remodeling at the NADH oxidation step in response to changes in EA availability. Furthermore, leveraging stepwise nitrate respiration, we identified the four NDH genes as members of the Crp and Arc regulons, which contribute to the remodeling of respiratory chains at the transcriptional level.

**Fig 7:**
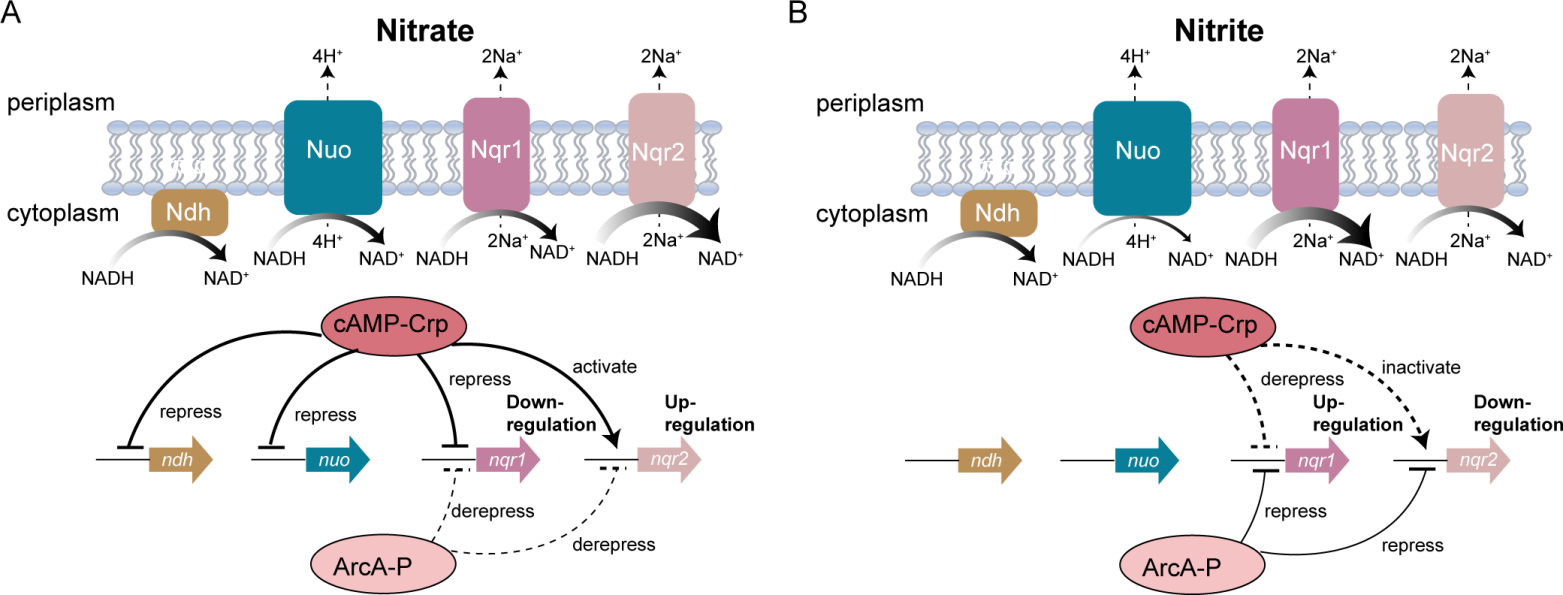
Proposed regulatory model of Crp and ArcA on the NDH genes in response to EA conversion from nitrate to nitrite. (A) When nitrate serves as EA, cAMP-activated Crp (cAMP-Crp) binds to the promoter region of *nqr1* operon as a repressor and while acting as an activator at the *nqr2* operon promoter. Although phosphorylated ArcA (ArcA-P) derepresses transcription of both *nqr* operons, its derepression effect on *nqr1* is counteracted by cAMP-Crp-mediated repression. The coordinated regulation by Crp and ArcA collectively downregulates *nqr1* expression and upregulates *nqr2* expression. Additional, cAMP-Crp binds to the promoter regions of *nuo* and *ndh*, repressing their transcription. (B) When nitrite serves as EA, cAMP-Crp derepresses the transcription of *nqr1* while inactivating *nqr2*. ArcA-P represses both *nqr* operons; however, its repression effect on *nqr1* is counteracted by cAMP-Crp-mediated derepression. This regulatory interplay results in upregulated *nqr1* expression and downregulated *nqr2* expression during nitrite respiration. Notably, no significant transcriptional regulation of *nuo* and *ndh* by Crp or ArcA was observed in nitrite respiration.

Sodium-pumping NDHs are prevalent in the *Shewanella* genus. Here, we demonstrate that the two *nqr* operons exhibit opposing transcriptional responses to EA conversion from nitrate to nitrite, a process critically governed by Crp. When nitrate serves as the EA, Crp directly represses *nqr1* while activating *nqr2* (Fig. 7A). Conversely, during nitrite utilization, Crp derepresses *nqr1* and inactivates *nqr2* (Fig. 7B). This regulatory switch ensures that only one *nqr* is robustly expressed during EA conversion in complete nitrate respiration. It is interesting to study the regulatory mechanism of Crp to discriminate the two homologous *nqr* operons.

In addition, the Arc regulatory system exerts modest repressive effects on both *nqr* operons during both nitrate and nitrite respiration (Fig. 7A and B), consistent with its canonical role in catabolic gene repression (64). Nitrate, a preferred high-redox-potential EA (5, 64), likely elevates the oxidized form of inner membrane quinones (65), mimicking conditions observed in the presence of oxygen (49) or high-potential electrode (38). This redox shift is sensed by the Arc system, triggering dephosphorylation of ArcA. Dephosphorylated ArcA derepresses both *nqr* operons (Fig. 7A). In contrast, when nitrite, a relatively low-redox-potential EA, is utilized, phosphorylated ArcA represses the transcription of both *nqrs* (Fig. 7B).

Nitrate serves as a preferred EA for bacteria due to its high standard redox potential. Our results showed that nitrate can be efficiently reduced by the *S. oneidensis* wild type and NDH triple mutants. In contrast, Δ*nuoN*Δ*nqrF1* exhibits significant defects in the respiration of high redox potential electrode and Fe(III)-NTA (26), processes that depend on the extracellular electron transfer pathway. These findings suggest that the redox potential of the utilized EA may not determine the preference for NDHs. The produced nitrite is a low redox potential EA. During nitrite respiration, all NDH triple mutants showed varying degrees of respiratory defects while retaining nitrite reduction capability, indicating that all four NDHs are involved in nitrite respiration. Interestingly, our results show that Nuo—the most efficient NDH—contributes least to nitrite respiration (Fig. 3B). Even though both Crp and the Arc system potentially regulate the transcription of *nuo*, this regulatory pattern cannot fully explain the limited contribution of Nuo to nitrite respiration. Notably, *S. oneidensis* possesses only one “uncoupling” Ndh. Our findings reveal that *ndh* also belongs to both the Crp and Arc regulons; however, its transcriptional level remains relatively stable during both nitrate and nitrite respiration. This consistent expression pattern suggests that *ndh* may play a crucial role in dissipating excess reducing power during anaerobic respiration.

In conclusion, this study demonstrates distinct NDH repertoire preferences during respiration of different EAs in *S. oneidensis*. Our findings corroborate that NDHs participate not only in extracellular insoluble EA respiration (38) but also in anaerobic respiration of periplasmic soluble EAs. The transcriptional change triggered by EA conversion mediates respiratory chain remodeling at the NADH oxidation step in *S. oneidensis*. Specifically, global regulators Crp and the Arc system coordinately control NDHs transcription. Crp plays a dominant role, particularly in mediating opposing transcriptional regulation between two *nqrs*. Our findings reveal that beyond their established regulation of terminal reductase genes, the global regulators Crp and Arc system also orchestrate upstream respiratory network complexity through NADH oxidation modulation. These regulatory mechanisms likely enable *Shewanella* species to dynamically remodel their respiratory chains for optimal survival in redox-stratified environments.

## SUPPLEMENTAL MATERIAL

Supplemental material spectrum.03324-24-s0001.docx Tables S1 to S3; Fig. S1 to S6.

ASM does not own the copyrights to Supplemental Material that may be linked to, or accessed through, an article. The authors have granted ASM a non-exclusive, world-wide license to publish the Supplemental Material files. Please contact the corresponding author directly for reuse.
