# Supplementary material for: Crp and Arc system directly regulate the transcription of NADH dehydrogenase genes in Shewanella oneidensis nitrate and nitrite respiration
Source: Microbiol Spectr. 2025 May 16;13(7):e03324-24. doi: 10.1128/spectrum.03324-24 (PMC12210985; doi:10.1128/spectrum.03324-24)
Supplement: Supplemental material — Tables S1 to S3; Fig. S1 to S6. [file spectrum.03324-24-s0001.docx]

| Strain or plasmid | Description | Reference |
| --- | --- | --- |
| *E. coli* |  |  |
| WM3064 | Donor strain for conjugation | W. Metcalf, UIUC |
| BL21(DE3) | Recombinant protein expression host strain | Novagen |
| *S. oneidensis* |  |  |
| MR-1 | Wild type | Lab stock |
| Δ*nqrF1* | *nqrF1* deleted mutant derived from MR-1 | This study |
| Δ*nqrF2* | *nqrF2* deleted mutant derived from MR-1 | This study |
| Δ*nuoN* | *nuoN* deleted mutant derived from MR-1 | This study |
| Δ*ndh* | *ndh* deleted mutant derived from MR-1 | This study |
| Δ*nuoN*Δ*ndh* | *nuoN* and *ndh* deleted mutant derived from MR-1 | This study |
| Δ*nuoN*Δ*nqrF1* | *nuoN* and *nqrF1* deleted mutant derived from MR-1 | This study |
| Δ*nuoN*Δ*nqrF2* | *nuoN* and *nqrF2* deleted mutant derived from MR-1 | This study |
| Δ*ndh*Δ*nqrF1* | *ndh* and *nqrF1* deleted mutant derived from MR-1 | This study |
| Δ*ndh*Δ*nqrF2* | *ndh* and *nqrF2* deleted mutant derived from MR-1 | This study |
| Δ*nqrF1*Δ*nqrF2* | *nqrF1* and *nqrF2*deleted mutant derived from MR-1 | This study |
| Δ*ndh*Δ*nqrF1*Δ*nqrF2* | *ndh*, *nqrF1* and *nqrF2* deleted mutant derived from MR-1 | This study |
| Δ*nuoN*Δ*nqrF1*Δ*nqrF2* | *nuoN*, *nqrF1* and *nqrF2* deleted mutant derived from MR-1 | This study |
| Δ*nuoN*Δ*ndh*Δ*nqrF1* | *nuoN*, *ndh* and *nqrF1* deleted mutant derived from MR-1 | This study |
| Δ*nuoN*Δ*ndh*Δ*nqrF2* | *nuoN*, *ndh* and *nqrF2* deleted mutant derived from MR-1 | This study |
| Δ*crp* | *crp* deleted mutant derived from MR-1 | This study |
| Δ*arcA* | *arcA* deleted mutant derived from M-1 | This study |
| Δ*crp*/*crp* | Complementation of Δ*crp* | This study |
| Δ*arcA*/*arcA* | Complementation of Δ*arcA* | This study |
| Plasmids |  |  |
| pHGM01 | Gene in-frame deletion used suicide vector, Ap^r^ Gm^r^ Cm^r^ | Jin et al. 2013 |
| pHGE-P*_tac_* | IPTG-inducible P*_tac_* expression vector | Luo et al. 2013 |
| pET-22b | His-tagged protein expression vector, Ap^r^ | Novagen |
| pMAL-c4x | MBP-tagged protein expression vector, Ap^r^ | NEB |

**Table S1 Bacterial strains and plasmids used in this study**

**Table S2 Primers used in this study**

| **Primer** | **Sequence** |
| --- | --- |
| Mutagenesis |  |
| *nuoN*-5O | GGGGACAAGTTTGTACAAAAAAGCAGGCTGGACTTGGCCGGTATTTTGC |
| *nuoN*-5I | AAGTGCGCCTAATCGCGTAGTTGGGAAGCGGAGAAAGTCA |
| *nuoN*-3I | CTACGCGATTAGGCGCACTTGGTCATCACTCTGGTGCAGG |
| *nuoN*-3O | GGGGACCACTTTGTACAAGAAAGCTGGGTTGGTGCGCAGTGATATGGAT |
| *ndh*-5O | GGGGACAAGTTTGTACAAAAAAGCAGGCTCTGACGTAACGCCCATCCTC |
| *ndh*-5I | AAGTGCGCCTAATCGCGTAGCTTAGAGGCAAGCGCCAAAC |
| *ndh*-3I | CTACGCGATTAGGCGCACTTTCAAAGGCACTTAGCGAGTCTT |
| *ndh*-3O | GGGGACCACTTTGTACAAGAAAGCTGGGTTTGCTGGCGAGGGGATTATAG |
| *nqrF1*-5O | GGGGACAAGTTTGTACAAAAAAGCAGGCTGGCTGGTATCAACCAAACGG |
| *nqrF1*-5I | AAGTGCGCCTAATCGCGTAGAGGTAAACCTCAAGCGGAGT |
| *nqrF1*-3I | CTACGCGATTAGGCGCACTTCGGCCGTGATCGGTATGTT |
| *nqrF1*-3O | GGGGACCACTTTGTACAAGAAAGCTGGGTCCATGAGCTAGCATCGCCTT |
| *nqrF2*-5O | GGGGACAAGTTTGTACAAAAAAGCAGGCTTGTTTAAGTTGCCACCAAGCG |
| *nqrF2*-5I | AAGTGCGCCTAATCGCGTAGTATCACCAGTAGGCACACCAC |
| *nqrF2*-3I | CTACGCGATTAGGCGCACTTCGAAAGCCTAGGGGTTGAGC |
| *nqrF2*-3O | GGGGACCACTTTGTACAAGAAAGCTGGGTCTAACTACAACACAGGGCGGT |
| *crp*-5O | GGGGACAAGTTTGTACAAAAAAGCAGGCTTGCCACGTTCACCCATATCTT |
| *crp*-5I | AAGTGCGCCTAATCGCGTAGAAAGTTGGGTCTGGTTTTGGC |
| *crp*-3I | CTACGCGATTAGGCGCACTTAGATCGTTGGTTGCTCCCG |
| *crp*-3O | GGGGACCACTTTGTACAAGAAAGCTGGGTGGCCATGGGCACGATGAATA |
| *arcA*-5O | GGGGACAAGTTTGTACAAAAAAGCAGGCTACCGATAAGTGCGCGATAGG |
| *arcA* -5I | AAGTGCGCCTAATCGCGTAGACGGGTAACGGCTTCATCTT |
| *arcA* -3I | CTACGCGATTAGGCGCACTTTACGCCAGAAATCATCGCCA |
| *arcA* -3O | GGGGACCACTTTGTACAAGAAAGCTGGGTCATTTGGGCGAAGCGGAAG |
| Complementation |  |
| pHGE-P*_tac_*-*crp*-F | CAATTTCACACAGGAGAGAATTCATGGCTCTGATTGGTAAGCCA |
| pHGE-P*_tac_*-*crp*-R | CCAAAACAGCCAAGCTTGGATCCTTAACGGGTACCATATACCACA |
| pHGE-P*_tac_*-*arcA*-F | CAATTTCACACAGGAGAGAATTCATGCAAAATCCGCACATTCTGA |
| pHGE-P*_tac_*-*arcA*-R | CCAAAACAGCCAAGCTTGGATCCTTAGTCTTCTAAGTTACCGCAGA |
| RT-qPCR |  |
| *nuoA*-F | CTGATGGGCGGTTTGGG |
| *nuoA*-R | CCGACGAGGATGGAGATGA |
| *ndh*-F | GTATTGTTGGTGCGGGTGC |
| *ndh*-R | AAGCCATCACGAGTCACTTCTT |
| *nqrA1*-F | CCGATGCTGAAATGGTGTT |
| *nqrA1*-R | CCGCTAAGCCGTCAAAGTC |
| *nqrA2*-F | GCCGCTGAGAATGACAACT |
| *nqrA2*-R | ACAATCCTCGGGTGCCTTA |
| *recA-F* | GCACTTGCTGCGGTATTGAG |
| *recA-R* | CCTGTAGAAATGGTCTCAACAT |
| EMSA/MST probe |  |
| P*nuoA*-F | GATGGTTTTTCCTAGAAGCGGT |
| P*nuoA*-R | TGGCGAACAGTTAAGACAGATC |
| P*ndh*-F | AATAACAACCTCAACAAACTCA |
| P*ndh*-R | GTGTCATTCCTGTTTTTGAATA |
| P*nqrA1*-F | ACTGAGTTAAATCTTGTTTAAAGTT |
| P*nqrA1*-R | ATCCACAAGCACTACGTTTTGA |
| P*nqrA2*-F | TCTATTTCTCCTACGGAAACTAT |
| P*nqrA2*-R | CATCACAAATATCAACCATGAAAA |

**Table S3 Characterization of multiple NDHs in *nuo*-containing *Shewanella* strains.** Number in each grid represents the isozyme(s) of corresponding type of NDH.

| **Strain** | **Nuo** | **Ndh** | **Nqr** |
| --- | --- | --- | --- |
| *Shewanella oneidensis* MR-1 | 1 | 1 | 2 |
| *Shewanella nanhaiensis* NR704-98 | 1 | 2 | 2 |
| *Shewanella woodyi* ATCC 51908 | 1 | 2 | 2 |
| *Shewanella* sp. E94 | 1 | 2 | 2 |
| *Shewanella* sp. D64 | 1 | 2 | 2 |
| *Shewanella* sp. MTB7 | 1 | 2 | 2 |
| *Shewanella hanedai* JCM 20706 | 1 | 2 | 2 |
| *Shewanella psychrophila* WP2 | 1 | 2 | 2 |
| *Shewanella* sp. UCD-KL12 | 1 | 3 | 2 |
| *Shewanella benthica* DB21MT-2 | 1 | 0 | 2 |
| *Shewanella eurypsychrophilus* YLB-08 | 1 | 1 | 2 |
| *Shewanella* sp. YLB-09 | 1 | 1 | 2 |
| *Shewanella violacea* DSS12 | 1 | 1 | 1 |
| *Shewanella benthica* KT99 | 1 | 1 | 1 |
| *Shewanella* sp. YLB-07 | 1 | 1 | 1 |
| *Shewanella psychropiezotolerans* YLB-06 | 1 | 1 | 1 |
| *Shewanella salipaludis* SHSM-M6 | 1 | 1 | 1 |
| *Shewanella* sp. AS16 | 1 | 1 | 1 |
| *Shewanella surugensis* DSM 17177 | 1 | 1 | 1 |

**Supplementary figures and legends**

**
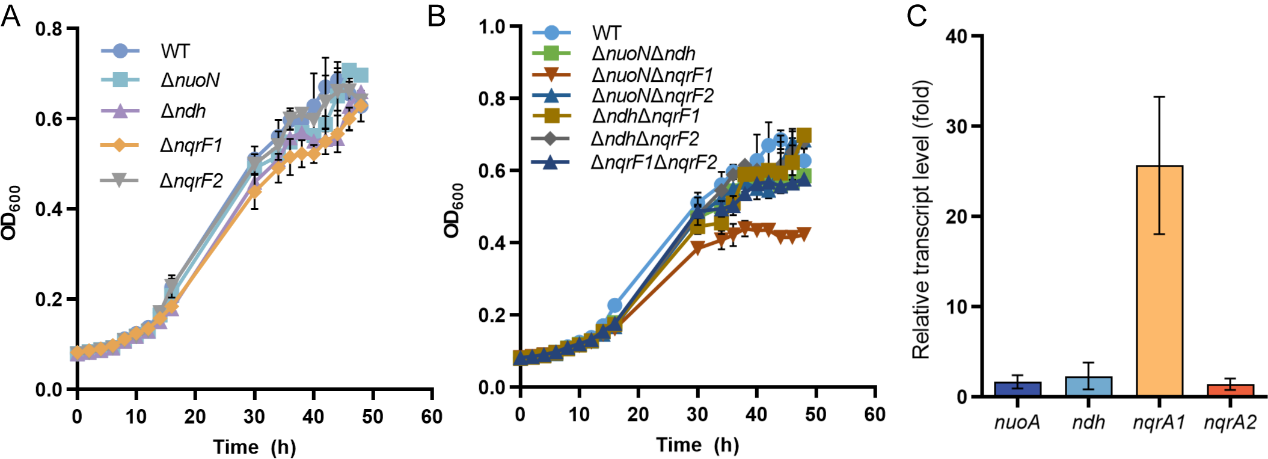
**

**Fig. S1 Characterization of NDH mutants and transcript levels of NDH genes under oxic conditions.** A. Growth of wild-type, Δ*nuoN*, Δ*ndh*, Δ*nqrF1* and Δ*nqrF2* strains in defined MS medium with lactate (20 mM) as sole electron donor under oxic conditions. B. Growth of wild-type, Δ*nuoN*Δ*ndh*, Δ*nuoN*Δ*nqrF1,* Δ*nuoN*Δ*nqrF2*, Δ*ndh*Δ*nqrF1*, Δ*ndh*Δ*nqrF2* and Δ*nqrF1*Δ*nqrF2* in defined MS medium with lactate (20 mM) as sole electron donor under oxic conditions. C. Relative transcript levels of *nuoA*, *ndh*, *nqrA1* and *nqrA2* in wild-type strain grown defined MS medium under oxic conditions. The error bar represents standard deviation of triplicate experiments. All experiments were carried out at least three times.

**
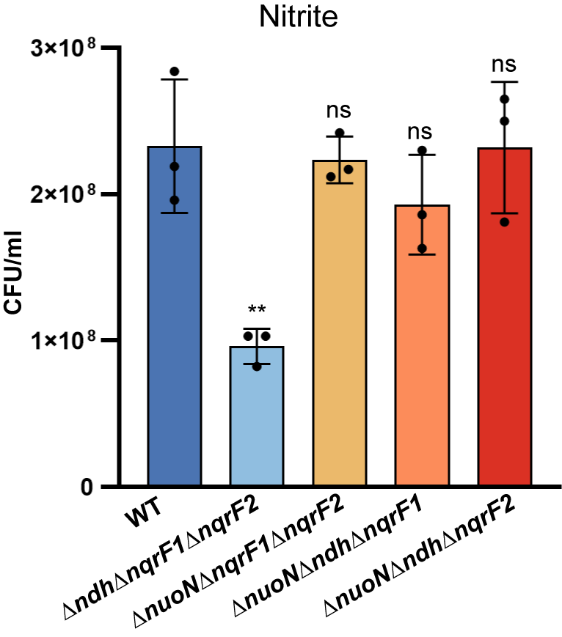
**

**Fig. S2 Maximum biomass supported by nitrite respiration under anoxic conditions.** *S. oneidensis* MR-1 (WT), Δ*nuoN*Δ*ndh*Δ*nqrF1*, Δ*nuoN*Δ*ndh*Δ*nqrF2*, Δ*nuoN*Δ*nqrF1*Δ*nqrF2* and Δ*ndh*Δ*nqrF1*Δ*nqrF2* were grown in defined MS medium with nitrite (5 mM) as sole EA under anoxic conditions. Cell densities of samples were measured by flow cytometer. The error bar represents standard deviation of triplicate experiments. A two-sided Student’s *t*-test was used to assess statistically significant differences (^**^, *p*<0.01; ns, *p*>0.05). All experiments were carried out at least three times.


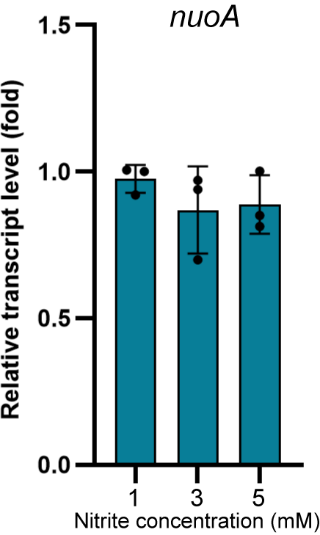


**Fig. S3 Transcription of *nuoA* did not response to nitrite concentrations.** Relative transcript levels of *nuoA* in wild-type strain grown in defined MS medium with nitrite at different concentrations as sole EA under anoxic conditions. The error bar represents standard deviation of triplicate experiments. All experiments were carried out at least three times.

**
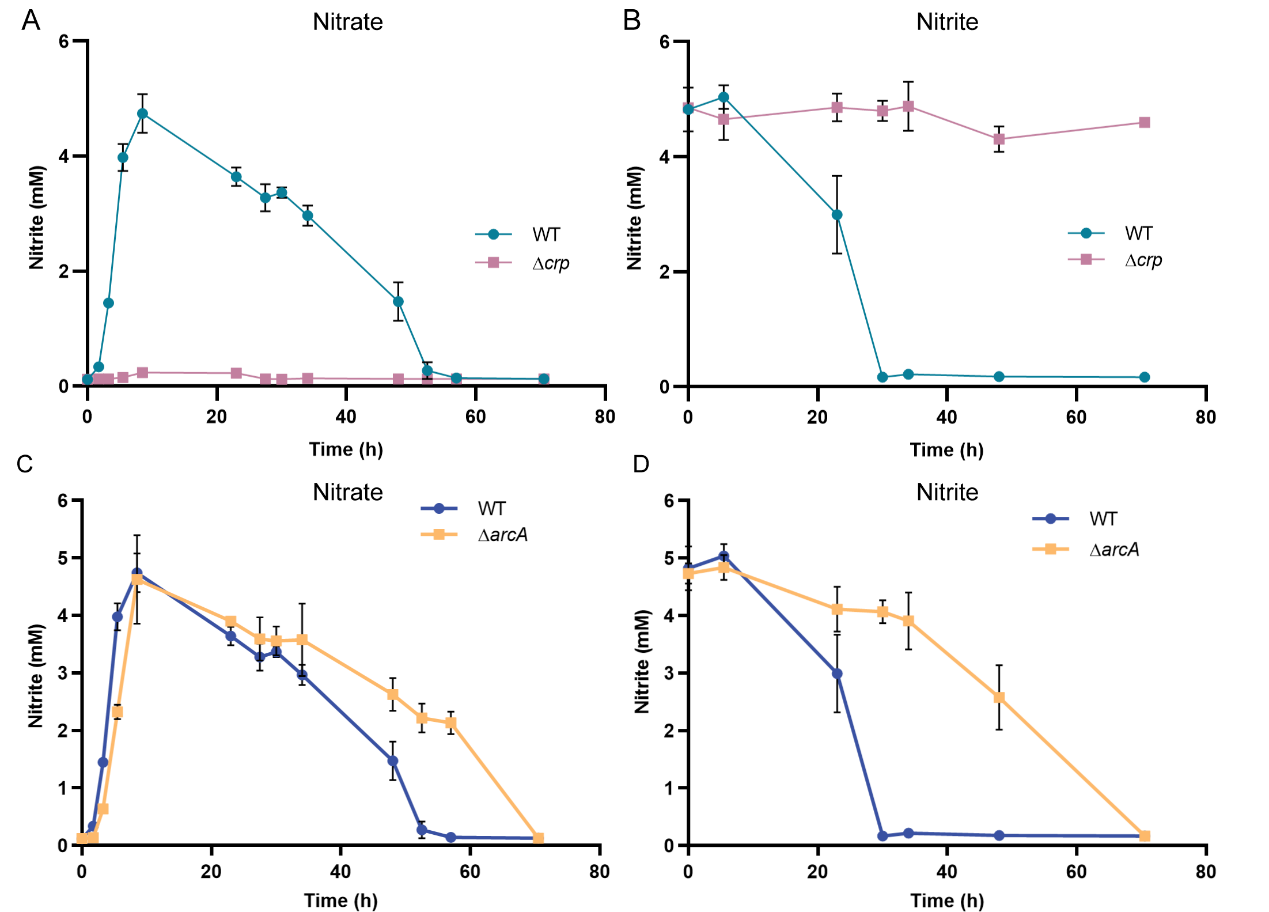
**

**Fig. S4 Nitrate and nitrite utilization by Δ*crp* and Δ*arcA.* A.** Nitrite concentrations in wild-type and Δ*crp* grown in defined MS medium with nitrate (5 mM) as EA under anoxic conditions. **B.** Nitrite concentrations in wild-type and Δ*crp* grown in defined MS medium with nitrite (5 mM) as EA under anoxic conditions. **C.** Nitrite concentrations in wild-type and Δ*arcA* grown in defined MS medium with nitrate (5 mM) as EA under anoxic conditions. **D.** Nitrite concentrations in wild-type and Δ*arcA* grown in defined MS medium with nitrate (5 mM) as EA under anoxic conditions. The error bar represents standard deviation of triplicate experiments. All experiments were carried out at least three times.


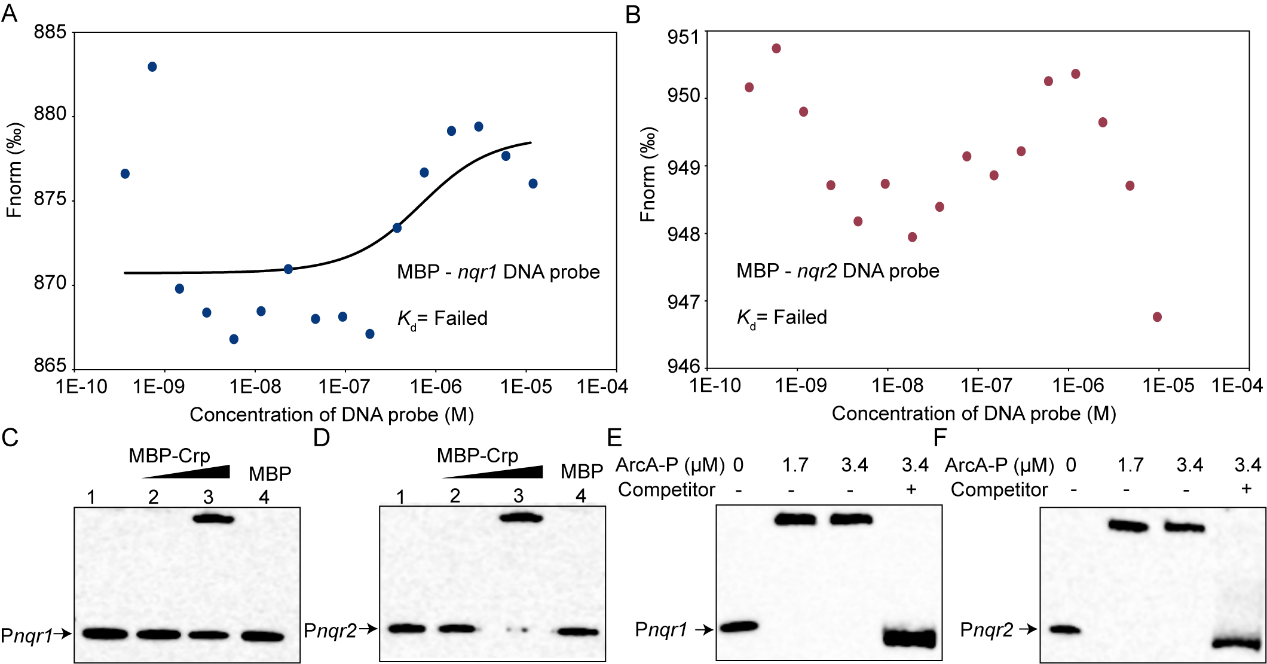


**Fig. S5 MBP-Crp and ArcA-P directly bind to the promoter regions of two *nqrs*. A.** MST analysis of purified MBP tag protein binding to *nqr1* promoter probe. **B.** MST analysis of purified MBP tag protein binding to *nqr2* promoter probe. **C,D.** EMSAs of MBP-Crp, activated by cAMP, titrated against biotin-labeled P*nqr1* (**C**) and biotin-labeled P*nqr2* (**D**). Lane 1, DNA probe (10 nM); lane 2 and lane 3, DNA probe (10 nM) mixed with increasing MBP-Crp (0.18 and 0.36 μM respectively); lane 4, DNA probe (10 nM) mixed with MBP (2.32 μM). **E,F.** EMSAs of ArcA-P titrated against biotin-labeled P*nqr1* (**E**) and biotin-labeled P*nqr2* (**F**). DNA probes (10 nM) were incubated with 0-3.4 μM ArcA-P in the absence (-) or presence (+) of a specific competitor (a 50-fold excess of unlabeled DNA probes) as indicated. Data presented are from at least three independent experiments and in all cases similar results were obtained.


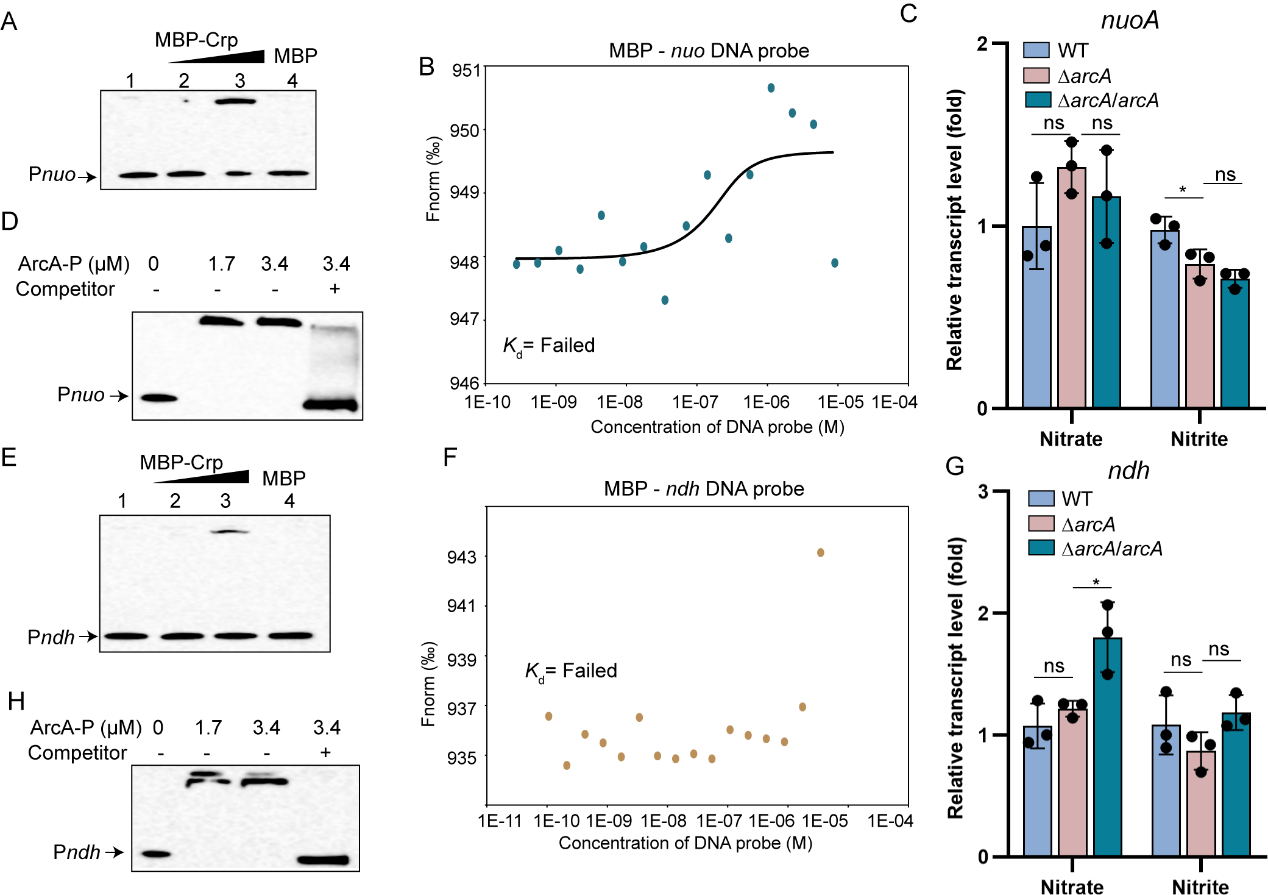


**Fig. S6 Crp and ArcA directly regulate the transcription of *nuo* and *ndh*. A,E.** EMSA of MBP-Crp, activated by cAMP, titrated against biotin-labeled P*nuo* (**A**) and biotin-labeled P*ndh* (**E**). Lane 1, DNA probe (10 nM); lane 2 and lane 3, DNA probe (10 nM) mixed with increasing MBP-Crp (0.18 and 0.36 μM respectively); lane 4, DNA probe (10 nM) mixed with MBP (2.32 μM). **B,F.** MST analysis of purified MBP tag protein binding to *nuo* promoter probe (**B**) and *ndh* promoter probe (**F**). MBP tag protein failed to bind to either promoter probe. **C,G.** Relative transcript levels of *nuoA* (**C**) and *ndh* (**G**) in wild-type, Δ*arcA* and its genetically complemented strain Δ*arcA*/*arcA* grown in M5 minimal medium with 5 mM nitrate or nitrite as EA in anaerobic conditions. The error bar represents standard deviation of triplicate experiments. **D,H.** EMSAs of phosphorylated ArcA (ArcA-P) titrated against biotin-labeled P*nuo* (**D**) and biotin-labeled P*ndh* (**H**). DNA probes (10 nM) were incubated with 0-3.4 μM ArcA-P in the absence (-) or presence (+) of a specific competitor (a 50-fold excess of unlabeled DNA probes) as indicated. A two-sided Student’s *t*-test was used to assess statistically significant differences (^*^, *p*<0.05; ns, *p*>0.05). Data presented are from at least three independent experiments and in all cases similar results were obtained.
